# Status of aflatoxin contamination in cow milk produced in smallholder dairy farms in urban and peri-urban areas of Nairobi County: a case study of Kasarani sub county, Kenya

**DOI:** 10.1080/20008686.2018.1547095

**Published:** 2018-11-27

**Authors:** Irene Kagera, Peter Kahenya, Florence Mutua, Gladys Anyango, Florence Kyallo, Delia Grace, Johanna Lindahl

**Affiliations:** a Department of Human Nutrition Sciences, Jomo Kenyatta University of Agriculture and Technology, Nairobi, Kenya; b Department of Animal and Human Health, International Livestock Research Institute, Nairobi, Kenya; c Department of Food Science and Technology, Jomo Kenyatta University of Agriculture and Technology, Nairobi, Kenya; d Department of Public Health, Pharmacology & Toxicology, University of Nairobi, Nairobi, Kenya; e Department of Public Health, Maseno University, Kisumu, Kenya; f Zoonosis Science Centre, Uppsala University, Uppsala, Sweden; g Department of Clinical Sciences, Swedish University of Agricultural Sciences, Uppsala, Sweden

**Keywords:** Aflatoxin M1, dairy production, food safety, ELISA, exposure, milk

## Abstract

**Introduction**: Milk consumption in Kenya supersedes other countries in East Africa. However, milk contamination with aflatoxin M1 (AFM1) is common, but the magnitude of this exposure and the health risks are poorly understood and need to be monitored routinely. This study aimed at assessing the awareness, knowledge and practices of urban and peri-urban farmers about aflatoxins and determining the levels of aflatoxin contamination in on-farm milk in a selected area within Nairobi County.

**Materials and methods**: A cross-sectional study was undertaken to assess aflatoxin contamination levels of milk in Kasarani sub-county. A total of 84 milk samples were collected from small-holder dairy farms and analyzed for AFM1 using Enzyme-Linked Immunosorbent Assay (ELISA).

**Results and Discussion**: Ninety nine percent of the samples (83/84) analysed were contaminated with AFM1. The mean aflatoxin level was 84 ng/kg with 64% of the samples exceeding the EU legal limit of 50 ng/kg. Whereas 80% of the farmers were aware of aflatoxin, there was no correlation between farmers’ knowledge and gender with AFM1 prevalence.

**Conclusion**: This study concludes that AFM1 is a frequent contaminant in milk and there is need to enhance farmers awareness on mitigation.

## Introduction

Aflatoxins (AF) are a group of mycotoxins produced as toxic secondary metabolites by fungus of *Aspergillus* species which is a grain storage organism. The optimal growth temperature is 25ºC with a minimum of 0.75 water activity, but already at 10-12ºC, the fungus starts to produce secondary metabolites [1]. *Aspergillus flavus* and *A. parasitucus* are the main producers of aflatoxins [2]. Among the well-known and naturally occurring types of aflatoxins are aflatoxin B1 (AFB1), aflatoxin B2 (AFB2), aflatoxin G1 (AFG1) and aflatoxin G2 (AFG2) [3]. The most prevalent aflatoxin and mostly found causing aflatoxicosis is AFB1, which is liable for carcinogenicity, chronic toxicity, acute toxicity, immunotoxicity and genotoxicity [1]. Metabolic derivatives of AFB1 and AFB2 are AFM1 and AFM2 respectively, and these metabolites can be present in urine and milk of animals fed on AFB1 contaminated feeds [3]. In vivo, metabolic oxidation of AFB2 and AFG2 to AFB1 and AFG1, respectively, makes them biologically active [4].

Prevalence of aflatoxins is high in latitudes between 40º N and 40º S, although the biggest health risks are in tropical developing countries where typical staple foods are affected by aflatoxins [3]. Aflatoxin contamination can occur along the production value chain starting from the field, during storage, and transportation and processing [5]. Among the staple foods affected by aflatoxins are cereals (wheat and maize), groundnuts, cassava, oilseeds (cotton, sunflower), fruits, wines, legumes, milk and milk products [6]. Major sources of human exposure to aflatoxins are groundnuts and maize because they are more susceptible to contamination and are frequently consumed worldwide [1]. Aflatoxin exposure routes are: ingestion of contaminated food, inhalation and skin contact. Aflatoxin can affect various organs and systems of animals in presence of other mycotoxins or individually [7,8].

Contamination of food with aflatoxins is a risk for both human beings and animals because apart from the grains that are mostly consumed, their by-products are frequently used as feeds, further, grasses and whole plants may be contaminated in the field and are a risk when used as forage for animals [1]. A batch of finished animal feed could be contaminated due to presence of only one contaminated ingredient, while feed shipments could be spoiled due to presence of aflatoxin contaminated feedstuff [5,7]. This spoilage has an effect in line with the international exchange of feed ingredients and animal feeds and the global trade [9].

Milk is important for both nutrition and development, especially in children, because of its diverse nutritional richness [10]. The dairy sector in Kenya contributes significantly to livelihoods of the many actors in the value chain. Milk is mainly from cattle, with goats and camel contributing less than 10% [11]. About 80% of milk is produced by peri-urban and rural smallholder dairy farmers [11]. The farms are more concentrated in urban and peri-urban areas where demand is high and access to market is easy.

Small-scale dairy farmers, especially those doing intensive farming, feed their dairy cows on commercial concentrates from uncertified agro-vet dealers, and the feed is often found contaminated with aflatoxins [12]. The risk of aflatoxin contamination through animal feeds will remain considering that most of the dairy cattle are kept in intensive farming systems and are fed on commercial concentrates that are often contaminated [13]. The complex situation where food safety and food security are weighed against each other in developing countries makes aflatoxin regulation difficult [14]. Contamination of milk with AFM1 is a public health issue, although the health effects of milk is not well understood [15]. This study gives baseline data on aflatoxin status in smallholder farms in Nairobi County, and can be used to support designing of strategies to mitigate the risk of exposure through milk produced in urban and peri-urban systems.

## Material and methods

### Study site selection

**Figure 1.**
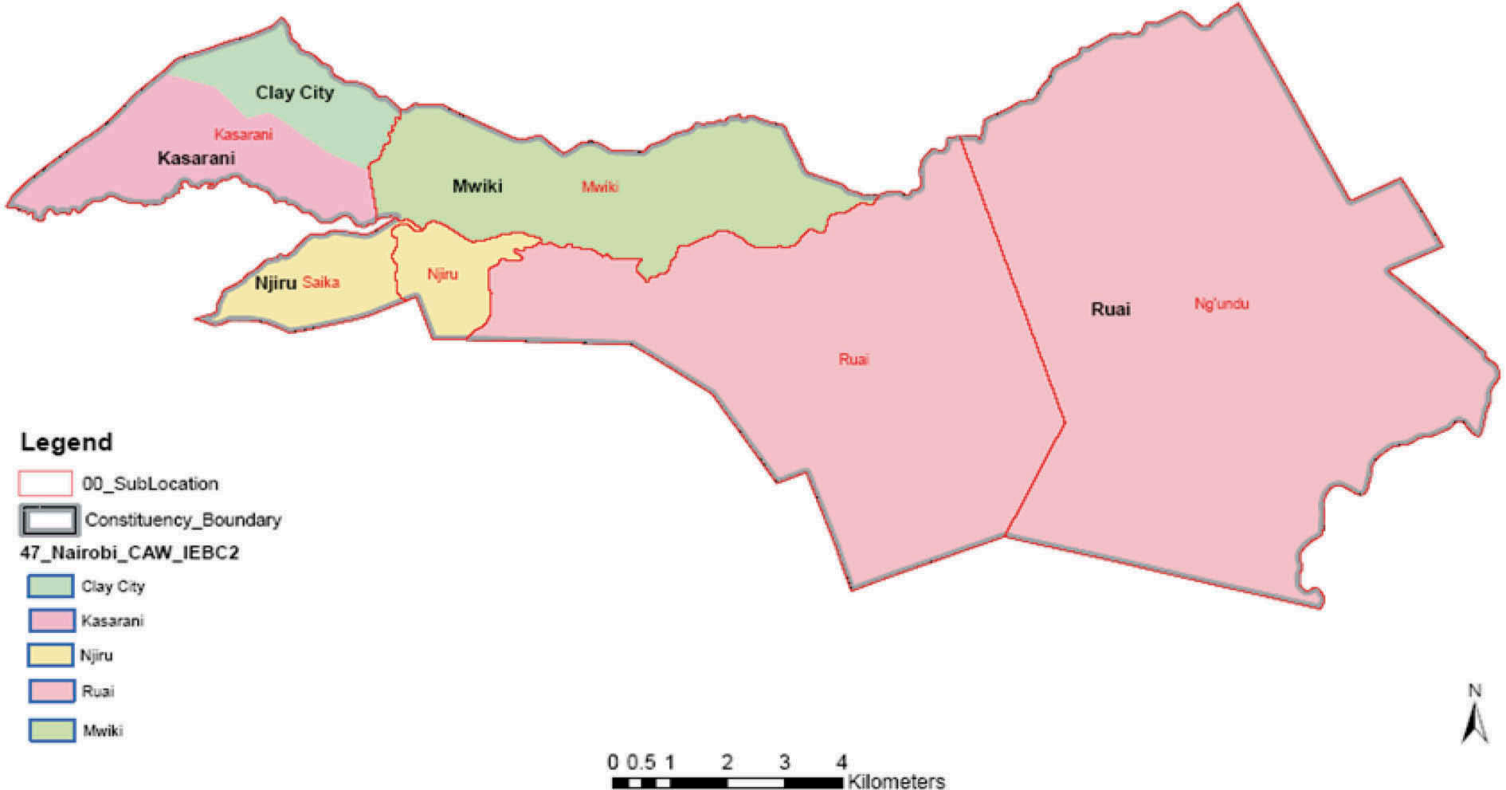
Map of Kasarani sub-county, Kenya, showing the study sites.

Kasarani sub-county was purposively selected for this survey since it has both urban and peri-urban areas with high number of smallholder farmers managing their cattle on zero-grazing system. Kasarani is one of the sub-counties in Nairobi County and has five wards, namely Kasarani, Mwiki, Clay city, Njiru and Ruai which were all included in the study (Figure 1).

### Sampling

The sub-county veterinary and livestock production department provided a list of dairy farmers which constituted the sampling frame, from which 100 farmers were randomly selected, using computer-generated random numbers, to participate in the survey. The sample size was calculated based on expected prevalence of milk samples above 50 ng/kg (AFM1) of 50% with a 10% precision [16]. Before the start of the survey, a one-day stakeholder participatory meeting was held to inform about the survey goals and seek consent to participate. The stakeholders included livestock extension officers, farmers, a Kenya Dairy Board representative and the county veterinary services director. The survey was done in the month of April 2017. Pre-testing of the questionnaire was done in one farm in Kasarani a week before the start of the survey. The pre-tested questionnaire was used to capture data on household and herd details, feeding and feed storage practices and farmers awareness on aflatoxins. A representative sample (50 ml) was taken from the bulked household milk. The samples were placed in a cool box that contained frozen ice packs to keep the milk cool during transportation to the laboratory at International Livestock Research Institute (ILRI) where it was frozen at −20 degrees awaiting analysis.

### Aflatoxin analysis

Detection and quantification of AFM1 was done using Aflatoxin M1 Low Matrix Enzyme-Linked Immuno-sorbent Assay (ELISA) kit (Helica Biosystem Inc., San Diego, USA). This ELISA detects AFM1 in concentration range of between 2 and 100 ng/kg. Samples that exceeded the highest standard (100 ng/kg) were diluted using skim milk (aflatoxin free) provided in the kit and re-tested in duplicates. An aliquot of 2 ml of each milk sample was centrifuged at 2000 rpm for 5 minutes to allow separation of the upper fatty layer. The upper fatty layer was removed and the lower plasma layer of the milk was used in the assay. Before use, all reagents provided in the kit were kept at room temperature for 30 minutes; standards and samples aliquots of 200 µl were dispensed in duplicate into appropriate wells coated with AFM1 antibodies. The plate was covered to avoid evaporation and to protect from excess UV light, and thereafter incubated for 2 hours at room temperature. Contents of the wells were discarded into a sink and each well washed three times using 250 µl of reconstituted wash buffer. After the washes, the wells were tapped face down on a layer of absorbent paper to remove residual wash buffer. After, 100 µl of conjugate was added to each well, the plate was covered and incubated for 15 minutes at room temperature. Additional washing was done three times and 100 µl enzyme substrate added to each well, followed by covering of the plate to avoid direct light and the plate incubated for 15 minutes. The reaction was stopped after the 15 minutes incubation by adding 100 µl stop solution which made the blue colour of the well contents turn yellow. Optical density (OD) of each well was read with a micro-plate reader at 450 nm using an air blank. The AFM1 level in each well was calculated using a logarithmic standard curve and the average of the duplicates used as the final results. This method has been previously described [12,17,18]

### Data analysis

Data were entered into Excel 2013 and exported to STATA Version 14.0 for analyses. Aflatoxin levels in milk were categorized into legal and high based on the laboratory results and the EU accepted level of 50 ng/kg. Qualitative data were summarized using graphs and frequency tables, while mean (±standard deviation), median and range values were determined for quantitative data. Chi square statistic was used to assess statistical associations of factors, for example, knowing if there is any significant association between gender of the respondent and aflatoxin status of the sampled milk (i.e. if below or above 50 ng/kg). A p-value level of 0.05 was used in assessment of statistical significance.

## Results

### Household characteristics

The total number of households surveyed was 100, corresponding to a response rate of 100%. The number of cattle owned by the interviewed households ranged between 1 and 60 animals with a median of 4 animals and an average 6 (sd 7.2) animals per household. On average, 47.50% of the owned animals were lactating and being milked at the time of the interview. The number of milked cows per household ranged between 1 and 18 with an average of 3 cows. Most (72%; n = 100) dairy farmers reared exotic breeds, a few kept crossbreeds (25%) and locals (3%). The exotic breeds included Holstein-Friesians, Guernsey, Jerseys, Ayrshire and Fleckvieh. Other livestock species kept were goats, sheep, poultry, pigs and donkeys.

### Respondents characteristic

**Figure 2.**
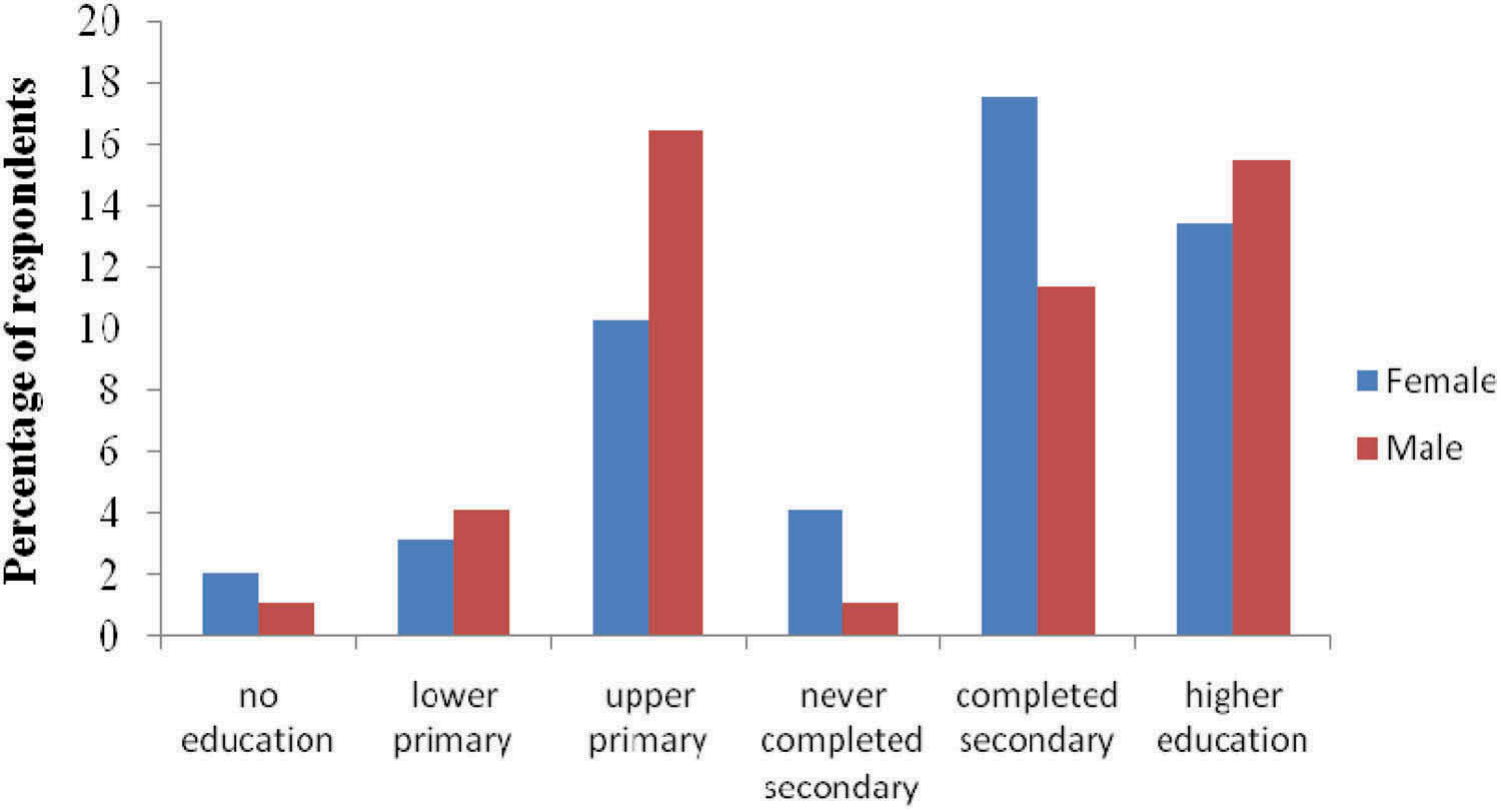
Education level of female and male respondents in Kasarani, Nairobi, Kenya.

The interviewed respondents aged between 20 and 85. The education level of the respondents varied from no education (3%) to the highest having reached to higher education (29%). More (18%) of the women respondents completed secondary school education as compared to the male respondents (11%). The male respondents who completed upper primary were more (16%) than female respondents (10%, Figure 2)

### Feeding practices

Overall, most of the respondents (89%, of which 49.4% were female and 50.6% were male) reported that they supplemented their animals with commercial concentrates. The management systems of the animals varied across households with majority of households practicing zero gazing (93%) and least number of households practicing pasture grazing (2%). Farmers who practiced pasture-grazing also supplemented their animals with either commercial or compounded feeds. The feed types used on the farms and their sources varied across the households, this included concentrates (99%), cut-carry pasture (97.9%), hay (98.8%) and silage (56.3%). The commonly purchased feed was concentrates (92.6%) while cut and carry was obtained mainly from own farms (52.8%, Table 1).

**Table 1.** Smallholder dairy farmers feeding practices in Kasarani, Kenya, and how the feed is sourced and stored.

		Origin of the product			
Feed type	%Households using	% On farm formulation	% Purchased	% stored on the floor	% stored on raised surface
Hay	98.78	11.54	88.46	22.06	77.94
Cut-carry-pasture	97.87	52.81	47.19	20	80
Concentrates	98.98	7.37	92.63	27.71	72.29
Silage	56.25	100	0	66.67	33.33

The different feed types used in the households were stored either on the floor or on a raised surface of which more than 50% of the hay (53%), cut-carry-pasture (60%) and concentrates (60%) were reported to be stored on a raised surface (Table 1).

### Milk production

Farmers milked their cows either twice or thrice per day depending with the cows’ milk production. The overall daily average milk production per household was 27 litres and average selling price of 64 Kenyan shillings (sd 8.3).

### Farmers’ knowledge, awareness about aflatoxin

**Figure 3.**
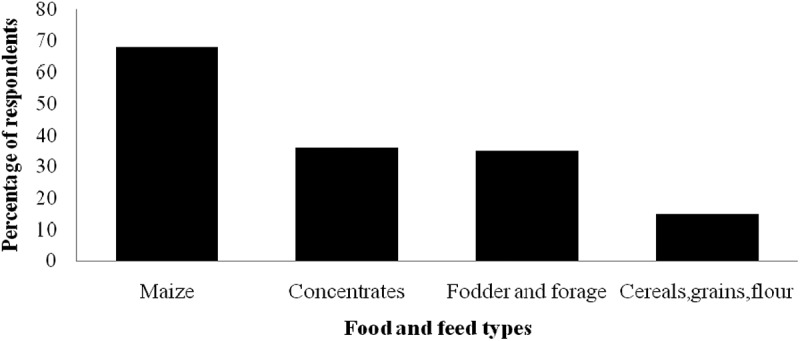
Percentage of dairy farmers in Kasarani, Kenya, that mention food and feed products that are prone to aflatoxin contamination.

Of the total respondents (100), women were more knowledgeable than men on issues regarding aflatoxins. Overall, 80% of the respondent said they had heard of aflatoxin of which 52% were women and 48% were men. Most of the respondents, 55%, gave the right information regarding aflatoxin, 45% of whom were men and 55% were women. Overall, 58% of the respondents said that presence of aflatoxin in some food and feed types pose danger to humans where 41.38% of these were male and 58.62% were women. According to the respondents, the food and feed types likely to be contaminated with aflatoxin included maize (68%), concentrates (36%), fodder & forage (35%), while 15% mentioned cereals in general, grains and flour (Figure 3).

### Aflatoxin M1, gender and awareness

During the time of survey, only 84 of 100 farmers selected had sellable milk in their household, therefore, a total of 84 milk samples were collected from all the five wards. Only 1 sample was below 2 ng/kg, the limit of detection. The mean concentration of AFM1 in the cow milk from Kasarani sub-county was 83.66 ng/kg (±64.68) with a maximum level of 255.96 ng/kg. Overall, 64% of the milk samples exceeded the EU limit of 50 ng/kg with a mean of 120.65 ng/kg, aflatoxin levels were higher in milk from households where the respondents were male (125.20 ng/kg) as compared to that of women respondents (116.09 ng/kg). The chi-square test showed no association between gender of the interviewed respondent and the milk having above or below 50 ng/kg (p = 0.77, Table 2). Though the majority of the respondents (64%) were aware about the effects of aflatoxin, milk from their households was more contaminated (mean of 90.35 ng/kg) when compared to contamination levels in households where respondents were not aware of aflatoxin effects (mean of 71.62 ng/kg). However, awareness was not significantly associated with milk having above or below 50 ng/kg (p = 0.12, Table 3).

**Table 2.** Aflatoxin M1 contamination in milk in female and male headed dairy farms in Kasarani, Kenya.

	Respondent gender	Number of samples	Mean aflatoxin M1 levels (ng/kg)	Standard deviation	Min	Max	Median
Overall	Female	41	82.37	61.98	2.06	212.82	85.01
	Male	43	84.88	67.88	<LOD	255.96	79.12
	**Total**	**84**	**83.66**	**64.68**	**<LOD**	**255.96**	**80.17**
<50 ng/kg	Female	14	17.34	18.18	2.06	48.91	7.80
	Male	16	16.84	16.12	<LOD	49.22	9.94
	**Total**	**30**	**17.08**	**16.81**	**<LOD**	**49.22**	**9.55**
≥50 ng/kg	Female	27	116.09	47.82	50.92	212.82	98.04
	Male	27	125.20	52.49	50.46	255.96	121.13
	**Total**	**54**	**120.65**	**49.95**	**50.46**	**255.96**	**107.52**

*p = 0.770. *LOD* Limit of detection (2 ng/kg).

**Table 3.** Aflatoxin M1 contamination in milk and the level of respondent awareness in Kasarani, Kenya.

	Status	Number of samples	Mean aflatoxin M1 levels (ng/kg)	Standard deviation	Min	Max	Median
Overall	Not aware	30	71.62	61.45	2.12	176.76	59.70
	Aware	54	90.35	66.02	<LOD	255.96	88.36
	**Total**	**84**	**83.66**	**64.68**	**<LOD**	**255.96**	**80.17**
<50 ng/kg	Not aware	14	16.76	17.03	2.12	48.91	9.94
	Aware	16	17.36	17.18	<LOD	49.22	9.09
	**Total**	**30**	**17.08**	**16.81**	**<LOD**	**49.22**	**9.55**
≥50 ng/kg	Not aware	16	119.63	42.22	58.76	176.76	121.24
	Aware	38	121.08	53.38	50.46	255.96	104.82
	**Total**	**54**	**120.65**	**49.95**	**50.46**	**255.96**	**107.52**

*p = 0.118. *LOD* Limit of detection (2 ng/kg).

## Discussion

This study reports on the occurrence of AFM1 in milk from urban and peri-urban smallholder dairy farms, as well as the knowledge of the farmers. In this study, more than 50% of the respondents were aware of aflatoxins, the sources, types of feed and food that can be easily contaminated with aflatoxin and the human health implications. However, none of the respondents reportedly knew that milk can also be contaminated with aflatoxins which was contrary to earlier report in Kenya [19,20].

Due to increased pressure on land for human settlement and absence of resources for farmers to run large scale dairy farming units, zero-grazing is the production system of choice for farmers in urban and peri-urban areas. In this study, majority of famers practice zero-grazing, thus relying more on purchasing pasture, fodder and concentrates with only a few making their own on-farm formulations. This is comparable to earlier reports in Kenya [21] in that farmers who formulated their own feeds used low-quality ingredients which could have been contaminated with aflatoxins due to poor storage at the source and on the farm.

This study showed that milk produced from urban and peri-urban dairy farms in Nairobi County is contaminated with AFM1. Most (64%) of the milk samples collected had AFM1 levels above the EU maximum limit of 50 ng/kg. The high levels of AFM1 in milk may be explained by the feeding practices. Though it was not in the scope of this study to determine the levels of AFB1 in the animal feeds, previous studies have documented their occurrence in the country [20,21]. The concentrations of AFM1 in this study were above those reported in Nakuru [21] but lower than those reported in others studies in Kenya [12,22] and Ethiopia [23]. This difference in the contamination levels can also be due to different cattle management systems, sources of feed and feed ingredients, and perhaps also feed storage conditions along the value chain.

Aflatoxin levels seemed higher in farms with more awareness, which may be confounded by ambitious or well-educated farmers having both higher producing cows, eating more concentrates, and also learning more. Thus it seems that the increased knowledge has not equipped farmers with the means to do on-farm mitigation. Aflatoxin in the dairy value chain is a public health problem and mitigation will need a OneHealth approach with involvement of stakeholders from the public health, agricultural and veterinary sectors.

## Conclusion

The milk in Kasarani sub-county is contaminated with AFM1 with most exceeding 50 ng/kg. The levels of contamination in milk in this study is of concern since the population in urban and peri-urban areas is highly exposed to AFM1, therefore, there is need to explore different mitigation strategies to control AFM1 in milk in urban and peri-urban areas. Feed ingredients and finished products should be thoroughly monitored to prevent cattle exposure to contaminated feeds which would lead to excretion of AFM1 in milk causing human exposure to through consumption. Aflatoxin control should start from the farm where feed raw materials are produced and along the dairy value chain. Use of mycotoxin clay binders should be explored at the farm and factory level; this would help to reduce contamination in feeds. More effort should be put in creating awareness on aflatoxin along the dairy value chain.
