# Supplementary material for: Status of aflatoxin contamination in cow milk produced in smallholder dairy farms in urban and peri-urban areas of Nairobi County: a case study of Kasarani sub county, Kenya
Source: Infect Ecol Epidemiol. 2018 Nov 27;9(1):1547095. doi: 10.1080/20008686.2018.1547095 (PMC6263096; doi:10.1080/20008686.2018.1547095)
Supplement: Supplemental Material [file ZIEE_A_1547095_SM5440.doc]

**Questionnaire used in the survey.**

**SURVEY CODE:**

**NAME OF THE ENUMERATOR _________________________**

**DATE: ___/___/2017**

**CHECK IF: Adequate farmer introduction has been done ____
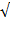
 | and Consent is granted _____
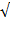
**

- 1. **Location of the farm**

| **County:** | **Ward:** | **Village:** | **GPS: Latitude** |
| --- | --- | --- | --- |
|  |  |  | **GPS: Longitude** |

**1.2 Household details**

| 1. **Respondent details** | **Gender:** | | **Age:** |  |
| --- | --- | --- | --- | --- |
|  | **Highest level of education:**  **[ ]** | | **Relationship to household head:: [ ]** | **Sources of income:**  **[ ] [ ] [ ]** |
|  | **1=never**  **2= primary (lower) 3=primary (upper)**  **4=secondary school (not completed)**  **5= secondary school (completed)**  **6= college / university** | | **0= respondent is household head**  **1= wife 2=husband**  **3=son 4=daughter**  **5=farm worker**  **6=other, specify** | **1=employed full time**  **2=employed casual**  **2= farming**  **3=other** |
| 1. **If respondent is not household head** | **Gender of household head:** | | **Age:** |  |
| 1. **Role in dairy farm** | **What is your role in feeding cattle?**  **[ ] [ ] [ ]** | **Role in selling milk**  **[ ] [ ] [ ]** | | **How responsible?**  **[ ] [ ] [ ]** |
|  | **1=Decided what feed to buy**  **2=Buy/acquire feed**  **3=Feed animals** | **1=Decide how much milk to sell**  **2=Decide where to sell milk**  **3=Sell milk**  **4=Control all money from sale**  **5=Control some money from sale** | | **1=Own cattle**  **2=Co-own cattle**  **3= involved with cattle**  **4=not involved cattle** |
| 1. **Previous training on dairy production: Yes/ No** | **What aspect of training was done :**  ***1=health 2= production 3= milk hygiene & safety 4= other*** | | **Specify which institution (or group) provided the training:** | **Which year was the training done :** |
| 1. **Presence of children up to 5 years of age** | **How many are they __________**  **What do the children eat – *list ingredients starting with the largest amount:***  **Morning……………………………………………………………………….**  **During the day………………………………………………………………**  **In the evening……………………………………………………………….** | | | |

**1.2 Who in the family is tasked with the following activities? (multiple numbers possible)**

| **Feeding of the animals** |  |
| --- | --- |
| **Milking of cows** |  |
| **Cleaning of the milking items** |  |
| **Selling of milk produced** |  |
| **Transporting milk to market** |  |

**CODE: 1= husband 2= wife 3= male worker 4=female worker 5= male relative 6= female relative 7= other, specify**

**1.3 Herd details**

**1.3.1 Details of cattle owned**

|  | **Adult males** | **Adult females** | | | | **Calves & weaners** |
| --- | --- | --- | --- | --- | --- | --- |
|  |  | **milked** | **Dry cows** | | **Heifers** |  |
| **Number on farm** |  |  |  | |  |  |
| **Specify breed(s) kept**  ***1= local 2=exotic, specify which one 3= crossbreeds*** | | | |  | | |
| **Management system**  ***1= pasture grazing 2=tethering 3= zero- grazing / cut and carry*** | | | |  | | |

**1.3.2How many of the following livestock species do you keep?**

**Goats: [ ] Sheep: [ ] Poultry: [ ] Donkeys: [ ] Pigs [ ] Other, specify: ___________ [ ]**

- - 1. **Animal Health**

| 1. **Have you ever encountered mastitis in your farm** | **[yes] [no] *If no, skip to 1.3.3 e*** |
| --- | --- |
| 1. **If yes, how often do you encounter mastitis in your herd** | **1= at least once in a week 2= at least once in a month 3= at least every two months 4= more rarely** |
| 1. **What do you do when your cow has mastitis?** | |
| 1. **What do you do to milk from a cow that has mastitis** | |
| 1. **If you treat with any medicine or drug, what do you use?** | |
| 1. **If you treat cow with any drug, what do you do with the milk from cow that is being treated** | |
| 1. **Do you check cows for mastitis: [ ] every milking [ ] Sometimes [ ] don’t do this** | |

**1.3.4 What do you use to perform the following milking tasks**

|  | **Does not do this** | **Cold water** | **Warm water** | **Soap** | **Disinfectant** | **Other- specify** |
| --- | --- | --- | --- | --- | --- | --- |
| 1. **To clean hands before milking** |  |  |  |  |  |  |
| 1. **To wash udder and teats before milking** |  |  |  |  |  |  |
| 1. **To wash udder and teats after milking** |  |  |  |  |  |  |
| 1. **Daily cleaning of milking shed** |  |  |  |  |  |  |
| 1. **More thorough cleaning of milking shed** |  |  |  |  |  |  |
| 1. **Ensure milking equipment is clean after milking** |  |  |  |  |  |  |

**1.3.5. Do you dry the teats before milking? If yes, with what? ________________________**

**1.4 Milk production**

**1.4.1 Amounts of milk produced**

| 1. **How often are the cows milked in a day** | | |
| --- | --- | --- |
| 1. **Indicate the amount of milk produced in a day (in LITERS) for the top 3 cows** | **Cow #1** |  |
|  | **Cow #2** |  |
|  | **Cow #3** |  |
| 1. **How much, in a day, is produced by the other milking cows** |  |  |

**1.4.2 The estimated total amount of milk produced a typical day, by all the cows on your farm, like yesterday, is __________ Liters**

**1.4.3 Description of sold milk**

| **What price is the milk sold at per liter** |  |
| --- | --- |
| **How long is the milk stored for before being sold (hrs)** |  |
| **How is the milk transported to market**  ***0= Customer or trader come to farm to pick milk 1= walk to deliver the milk 2= use own bicycle 3= use public vehicle 4= use own vehicle*** |  |

**1.4.4 Who buys the milk that you produce (indicate number and quantity sold per day)**

| **Who buys** | **Quantity (L) sold per day** |
| --- | --- |
| **1. neighbor for home use** |  |
| **2. milk trader** |  |
| **3. hotels or shops** |  |
| **4. bulking Centre** |  |
| **5. other : __________** |  |

**1.4.5 Of the total milk kept by the household how much is.**

|  | **Consumed raw** | **Consumed boiled** | **Processed e.g. fermented** |
| --- | --- | --- | --- |
| **Estimate of amounts in liters yesterday** |  |  |  |

**1.4.6 Describe how fresh milk is stored before being consumed within farm**

**1.4.7 Does your milk ever get spoilt? If yes, what do you do to spoilt milk?**

**1.5 Feeding**

**1.5.1 Feeding and feed storage practices**

| **Feed type used by the farmer** | | | | | | |
| --- | --- | --- | --- | --- | --- | --- |
|  | **Open grazing** | **Hay bales** | **Cut-carry- pasture** | **Concentrates/compound feed** | **Silage** | **Molasses** |
| **Does this feed option apply (check)** |  |  |  |  |  |  |
| **What is the source of the feeds** | **NA** |  |  |  |  |  |
| **How is the feed stored** | **NA** |  |  |  |  |  |
| **Do you routinely monitor the condition of your feed during storage, for any spoilage *[yes] [no]*** | **NA** |  |  |  |  |  |
| **If yes, what conditions do you routinely monitor for during storage** | **NA** |  |  |  |  |  |
| **What actions would you take if you noticed your stored feed had molds** | **NA** |  |  |  |  |  |

**CODES**

**Source of feeds: 1= on farm formulations 2= purchased, specify price per unit 3= other sources, specify**

**How is the feed stored: 1= on the floor 2= on raised surfaces 3= other, specify**

**Storage conditions routinely monitored for: 1= moisture 2= warmth 3= ventilation 4= mold growth 5= dryness 6= pests / animal**

**Actions If stored feed had molds: 1= dispose the feed 2= still give animals the feed 3= mix with good feed**

**1.5.2 Observe if there is a feed storage facility within the farm, if not, ask and describe how / where the feed is stored**

***1.5.3 Supplementation with concentrate / commercial feeds*** (applies to what the farmer is using at the time of the study visit)

| **Feed type** | **Description of the feed (brand)** | **Quantities (kg) given per day/ cow** | **What do you use to measure the portion you feed** | **How is the feed provided to the animals** |
| --- | --- | --- | --- | --- |
|  |  |  |  |  |
|  |  |  |  |  |
|  |  |  |  |  |
|  |  |  |  |  |

**CODE**

**Description of feed: 1=bought commercial (specify brand type) 2= on farm formulation (specify ingredients)**

**How is the feed provided to animals: 1= alone 2= as a mix with other feeds**

**1.5.4 What else do you routinely add to your feeds?**

**1.6 Awareness about molds and aflatoxins**

**1.6.1 Have you ever seen mold on cattle feed, in your farm______________ [yes] [no]**

**1.6.2 If yes, do you think it has any impacts on cattle and if so what impact(s)**

**1.6.3 Have you heard of aflatoxins _________ [yes] [no]**

**1.6.4 If yes, what are they?**

**1.6.5 If yes to 1.6.3 above, which products (food types, feed types etc.) would you expect to be easily contaminated with aflatoxins? _____________________________________________________________________________________**

**1.6.6 Do you think the presence of aflatoxins in these foods pose any danger to humans? Which danger(s)?**

**1.7 If you get invited to participate in the next stage of the study, we will facilitate getting you different things to help in your milk production, some of which may cost you some money to acquire.**

| **Mazzicans** | **Milk container (capacity 10L) that facilitates mastitis detection during milking, allows for safe storage and transportation of milk, reducing spoilage in the milk chain** |
| --- | --- |
|  | **1.7.1 Would you be willing to acquire and use such containers** |
|  | **1.7.2 How much (ksh) would you be willing to pay for such a container** |
| **Aflatoxin binders** | **A form of clay that, when mixed with feeds, sticks to any aflatoxins present so that much of the aflatoxins pass out in the cattle faeces and can’t enter milk** |
|  | **1.7.3 Would you be willing acquire and use aflatoxin binding agents** |
|  | **1.7.4 How much (per KG) would you be willing to contribute to get a substance that will reduce aflatoxins for your cow** |

**SAMPLING OF MILK**

| **Collect 2 x 40 ml in sterile falcon tubes from milk that is meant for household consumption or for sale** | |
| --- | --- |
| **Indicate the approximate time the sampled milk was milked** |  |
| **Indicate if the sampled milk has been treated in any way, e.g. by boiling, chilling** |  |
| **Indicate the approximate date and time when the sample is collected** |  |

**Would you be willing to participate in a future program to make your milk safer? If yes, please give us your name and phone number. Note that you can change your mind and say no when invited to participate.**

**Name: _______________________________________________**

**Phone Number: _____________________________________________**

**….THANK YOU VERY MUCH FOR YOUR TIME, WE VALUE YOUR INPUTS….**
